# Effects of Embodiment in Virtual Reality for Treatment of Chronic Pain: Pilot Open-Label Study

**DOI:** 10.2196/34162

**Published:** 2024-02-16

**Authors:** Adam Saby, Anthony Alvarez, David Smolins, James Petros, Lincoln Nguyen, Michael Trujillo, Oytun Aygün

**Affiliations:** 1 Department of Emergency Medicine Occupational Health Division University of California Los Angeles Los Angeles, CA United States; 2 Karuna Labs San Francisco, CA United States; 3 Remedy Medical Group San Mateo, CA United States; 4 Allied Pain and Spine San Jose, CA United States; 5 Karuna Labs New York, NY United States

**Keywords:** centralized pain, dicentralized pain, digital therapeutics, visual analog scale, Fear-Avoidance Beliefs Questionnaire, Oswestry, Oswestry Low Back Pain Disability Questionnaire, Pain Catastrophizing Scale, Patient Health Questionnaire, sensorimotor, virtual reality, chronic pain, pain, rehabilitation

## Abstract

**Background:**

Chronic pain has long been a major health burden that has been addressed through numerous forms of pharmacological and nonpharmacological treatment. One of the tenets of modern medicine is to minimize risk while providing efficacy. Further, because of its noninvasive nature, virtual reality (VR) provides an attractive platform for potentially developing novel therapeutic modalities.

**Objective:**

The purpose of this study was to determine the feasibility of a novel VR-based digital therapy for the treatment of chronic pain.

**Methods:**

An open-label study assessed the feasibility of using virtual embodiment in VR to treat chronic pain. In total, 24 patients with chronic pain were recruited from local pain clinics and completed 8 sessions of a novel digital therapeutic that combines virtual embodiment with graded motor imagery to deliver functional rehabilitation exercises over the course of 4 weeks. Pain intensity as measured by a visual analog scale before and after each virtual embodiment training session was used as the primary outcome measure. Additionally, a battery of patient-reported pain questionnaires (Fear-Avoidance Beliefs Questionnaire, Oswestry Low Back Pain Disability Questionnaire, Pain Catastrophizing Scale, and Patient Health Questionnaire) were administered before and after 8 sessions of virtual embodiment training as exploratory outcome measures to assess if the measures are appropriate and warrant a larger randomized controlled trial.

**Results:**

A 2-way ANOVA on session × pre- versus postvirtual embodiment training revealed that individual virtual embodiment training sessions significantly reduced the intensity of pain as measured by the visual analog scale (*P*<.001). Perceived disability due to lower back pain as measured by the Oswestry Low Back Pain Disability Questionnaire significantly improved (*P*=.003) over the 4-week course of virtual embodiment regimen. Improvement was also observed on the helplessness subscale of the Pain Catastrophizing Scale (*P*=.02).

**Conclusions:**

This study provides evidence that functional rehabilitation exercises delivered in VR are safe and may have positive effects on alleviating the symptoms of chronic pain. Additionally, the virtual embodiment intervention may improve perceived disability and helplessness of patients with chronic pain after 8 sessions. The results support the justification for a larger randomized controlled trial to assess the extent to which virtual embodiment training can exert an effect on symptoms associated with chronic pain.

**Trial Registration:**

ClinicalTrials.gov NCT04060875; https://clinicaltrials.gov/ct2/show/NCT04060875

## Introduction

Chronic pain is a major health care problem worldwide**.** About 20% of the US [1-3] and European [4-6] populations have been reported to experience chronic pain. Chronic pain is associated with anxiety, depression, and other psychological disorders [7-13], as well as impairment in executive functions [14] and decision-making [15]. Opioids are one of the most common treatment strategies for chronic pain despite the potential for abuse, dependence, misuse, and accidental overdose [16,17]. Chronic pain is a consequential burden on society due to impairment of personal well-being, loss of productivity, and opioid use and dependence. Opioid addiction has been declared a public health emergency by the US Health and Human Services due to the potential misuse by more than 2 million people and more than 47,000 deaths annually [18].

While the specific neural mechanisms of chronic pain are variable and elusive, it has been proposed that a maladaptive neuroplasticity in the anterior cingulate cortex (ACC) occurs, leading to the long-term potentiation in neural circuits associated with the ACC [19]. The ACC has been shown to elicit rate, spatial, and temporal coding that is specific to the anticipation of pain [20]. The ACC shares connections with the periaqueductal gray (PAG), which is the primary control center for descending pain neuromodulation. The disruption of ACC and PAG circuit activation may impair the ability of the descending pain suppression pathway to modulate pain in patients with chronic pain [21]. Long-term potentiation in the ACC may alter nociceptive processing, resulting in hyperalgesia and hyperpathic anticipation of painful events. The ACC and PAG also interact with the amygdala of the limbic system, which has been suggested to modulate pain pathways [22-27]. Through the involvement of the amygdala in pain pathways, the emotions such as stress and fear modulate pain [24,27-29].

Fear-avoidance models of pain suggest that attitudes and beliefs about pain are predictors of pain-related disability, as pain catastrophizing beliefs cause behavioral avoidance [30-37]. Pain catastrophizing has been defined as an exaggerated negative attitude toward pain experience [38]. Pain catastrophizing has been linked to the transition of acute pain into chronic pain [30,39], as well as a negative attitude toward medical procedures in patients with chronic pain [40,41]. Interventions aiming to develop adaptive psychological attitudes and strategies are suggested to reduce the level of pain catastrophizing and thus having a positive impact on patient outcomes [32,42].

Virtual reality (VR) has emerged as a novel technology for the treatment of pain, showing promise as a treatment strategy for chronic pain, burn pain, acute pain, and reducing the intensity of experimentally induced pain [43-50]. There are 2 strategies of VR that may promote analgesic effects: distraction therapy and immersiveness [47,48,51]. Distraction therapy temporarily diverts the attention of a user, which leads to reduced intensity of pain. Immersiveness, also known as embodiment, is the phenomenon by which a person identifies with and develops a sense of ownership over a body part that is not their own. Embodiment allows a user to interact with an artificial environment and receive altered visual feedback associated with the movement of a virtual avatar, which is controlled by the user’s manipulation of the VR hardware. Immersiveness in VR may activate premotor and somatosensory circuitry associated with the body parts that are embodied [52]. Thus, there may be a reorganization of the sensorimotor representations within the central nervous system in a way that the perception of a painful limb is modified, resulting in people perceiving the limb as less painful [48,53-55]. Furthermore, virtual embodiment has been shown to influence pain-free range of motion in patients with unilateral chronic shoulder pain [49].

Digital rehabilitation interventions could have multidimensional, biopsychosocial effects for managing pain [56,57]. In patients with chronic neck pain using digital rehabilitation therapy, disability and range of motion was improved [58,59], suggesting that VR interventions are promising for treatment of chronic pain. Multiple sessions of Fear Avoidance Beliefs Training through a rehabilitation medical device have shown improvements in the Pain Catastrophizing Scale (PCS), along with reductions in pain intensity and mobility impairment and disability [49]. Active coping strategies, such as exercise in chronic pain, were shown to have better outcomes in pain-related disabilities compared to passive strategies such as taking medication or resting [60]. Immersiveness in VR could potentially provide the patients with a more active participation in their treatment for chronic pain.

Chronic pain is a health care problem worldwide. There is a compulsory need for alternative, noninvasive, and nonaddictive therapeutics for treating chronic pain [61]. The purpose of this study was to determine the feasibility of a novel VR-based digital therapy for the treatment of chronic pain. The safety and feasibility of an intervention through embodiment in VR to treat chronic pain was assessed. In this study, 24 patients with chronic pain received a novel VR-based functional rehabilitation program (Karuna Virtual Embodiment Training [KVET]; Karuna Labs, Inc) over the course of 4 weeks, with 2 sessions per week. Self-perceived pain intensity rating, pain catastrophizing, and self-perceived disability were measured. The measurements were anonymized for analysis. We hypothesized that gradual exposure of patients to functional rehabilitation in immersive therapy in VR can be used to help patients overcome pain-related fears and catastrophizing beliefs. We hypothesized that an immersive VR therapy would have a positive impact on patient outcomes.

## Methods

### Ethical Considerations

This study’s protocol was approved by Advarra's institutional review board (reference number: Pro00026459) and conducted in accordance with the ethical standards of the Declaration of Helsinki.

### Study Procedure

This trial was registered at ClinicalTrials.gov (NCT04060875). In total, 24 adult participants that were diagnosed with chronic (for 3 months or longer) lower back or chronic upper extremity pain were recruited from local pain clinics over the normal course of business. Individuals with a history of severe mental illness, including schizophrenia, bipolar disorder I or II, and posttraumatic stress disorder; a history of susceptibility to seizures per the participant’s reporting; and pregnant women were not included in this study. Interested participants received information regarding virtual embodiment training and were scheduled for an intake visit. During the intake visit, participants completed a written informed consent and then were administered initial outcome measures (patient-reported pain questionnaires). To assess potential adverse events associated with VR, the Simulator Sickness Questionnaire (SSQ) was administered to all potential participants. The SSQ helps identify the risk of nausea and dizziness in VR [62]. In this study, no patients exceeded the exclusion threshold on the SSQ, and none were excluded from participation on this basis. Qualified participants received information regarding VR therapy and were scheduled for an intake visit. During the intake visit, all recruited candidates completed a written informed consent, and baseline instruments for outcome measures were administered. All recruited participants completed this study.

### Intervention

Virtual embodiment sessions were administered on an HTC Vive (HTC Corporation) VR head-mounted display (110º field of view, 1080 × 1200 pixels/eye, and 90 Hz refresh). The HTC Vive hand controllers and Vive trackers were used to provide an immersive VR experience. The HTC Vive accurately tracks limb and trunk position and movement and delivers an immersive experience where a virtual avatar is controlled by manipulating the position of the hand controllers, Vive tracker, and head-mounted display.

**Figure 1:**
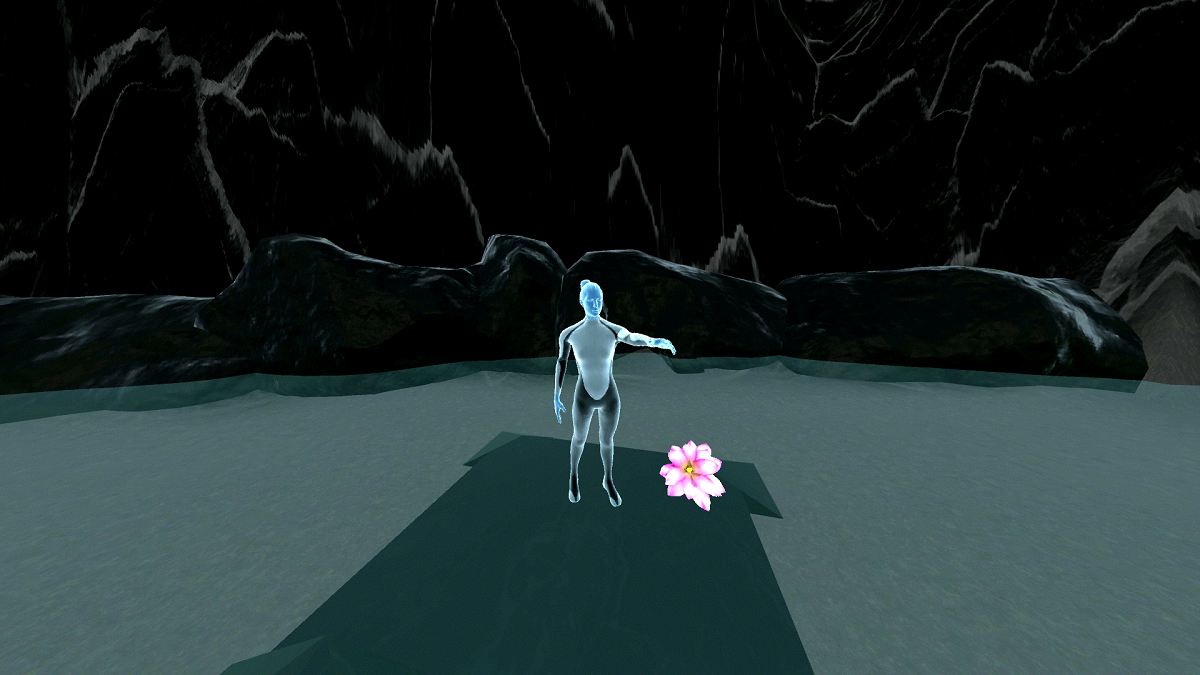
An example of a virtual avatar. In this exercise, the patient is participating in an immersive functional movement exercise. The figure shows a third-person view of what a patient would be doing in an exercise. In the exercise, participants grasp floating orbs in an immersive virtual reality experience. Shoulder flexion, scaption, and internal and external rotation are measured in the experience.

Patients received biweekly sessions of virtual embodiment training over the course of 4 weeks. Visual analog scale (VAS) was administered before each session to obtain an indication of baseline pain intensity levels. Patients were then administered virtual embodiment training. Virtual embodiment training sessions progressed from 20 minutes in the initial session to 45 minutes in later sessions. Following each virtual embodiment training session, the VAS was readministered to determine whether individual sessions provide reductions in self-perceived pain intensity. KVET consists of exercises in an embodied VR experience that are designed based on the principles of graded motor imagery (GMI). In this study, patients with upper-extremity chronic pain engaged in the 4 virtual embodiment training modules: laterality, motor imagery, “mirroring,” and predictive coding. Laterality training consisted of movements mirrored from one side to the other by an avatar in VR. In the motor imagery module, the participant experienced a first-person perspective view of virtual avatar limbs moving while being instructed to imagine the movement is happening in his or her own body. In the mirroring module, a participant’s healthy limb is used to produce movements that appear to be occurring in the painful limb. In the predictive coding module, participants were surrounded by floating orbs in a virtual environment (Figure 1). The orbs were placed at random locations within arm’s reach of the participant. Participants were instructed to reach and grab the orbs promoting upper-extremity movement. A description of rehabilitation exercises administered in KVET for patients with chronic low back pain has been previously published [49].

### Measures

Self-perceived pain intensity served as the primary outcome measure and was evaluated before and after each virtual embodiment training session using a VAS for pain. Participants were asked to denote pain using a 10-cm line with the left end representing no pain and the right indicating the worst possible pain. Participants would then check off the region of the line they believed accurately represented their current pain. The distance between the left most part of the 10-cm line and the participants check mark was then measured using a standard ruler. This instrument is standard for tracking pain [63]. A 2-way ANOVA on session (1-8) was used to assess differences in VAS score as a function of session and clinical intervention.

To assess the appropriateness of outcome measures on a larger scale, exploratory outcome measures were administered for pain, physical functioning, mental functioning, and disability. Patient-reported pain questionnaires were administered before the first session of virtual embodiment training and after the last session. The validated instruments included the Fear-Avoidance Beliefs Questionnaire (FABQ), Oswestry Low Back Pain Disability Questionnaire (ODI), PCS, and the Patient Health Questionnaire. The FABQ is a questionnaire measuring the effect that pain has on daily life activities [64]. The ODI, frequently used in research of physical therapy efficacy for treatment of lower back pain [65,66], was applied to the subset of participants whose diagnoses included low back pain. The PCS includes 3 subset scales: rumination, magnification, and helplessness [67]. Due to the ordinal nature of subjective pain questionnaires and the likeliness of nonnormal distribution, a Wilcoxon signed rank test was used to assess the difference in the subjective pain questionnaire score before and after clinical intervention.

## Results

### Self-Perceived Pain Intensity

Self-perceived pain intensity as measured by the VAS improved as a function of individual virtual embodiment training sessions. A 2-way ANOVA on session (1-8) × pre- versus posttraining on the VAS revealed a significant main effect of KVET therapy sessions (*F*_1,8_=14.246; *P*<.001; Cohen *d*=0.504). There was no significant main effect of the number of sessions (*P*=.85).

### Patient-Reported Pain Questionnaires

Pre- and posttraining FABQ work and physical activity scores were recorded for 14 participants. A Wilcoxon signed rank test revealed no significant difference between pre- and post-KVET scores for FABQ-work (*Z*=–0.629; *P*=.57; mean pretest score 26.14, SD 13.04; mean posttest score 24.86, SD 13.07) nor FABQ-physical activity (*Z*=–1.480; *P*=.15; mean pretest score 15.07, SD 7.08; mean posttest score 13.21, SD 7.16).

Pre- and posttraining Patient Health Questionnaire scores of the 4-week course were recorded for 10 participants. A Wilcoxon signed rank test revealed no significant difference between pre- and post-KVET scores (*Z*=–1.262; *P*=.23).

Pre- and posttraining ODI scores for the 4-week course were recorded for the population of participants experiencing low back pain. A Wilcoxon signed rank test revealed a significant improvement on how patients rated their disability associated with low back pain after KVET (*Z*=–2.819; *P*=.003; mean pretest score 23.07, SD 9.42; mean posttest score 19.73, SD 6.99). A post hoc measurement of effect size revealed a medium treatment effect of KVET (Cohen *d*=0.401).

Pre- and posttraining PCS scores for the 4-week course were recorded for 15 participants. A Wilcoxon signed rank test revealed a significant improvement of pain catastrophizing in the helplessness category (*Z*=–2.254; *P*=.02; mean pretest score 11.60, SD 6.62; mean posttest score 9.13, SD 5.10). A post hoc measurement of effect size revealed a medium treatment effect of KVET (Cohen *d*=0.418). No significant difference was observed for PCS rumination (*Z*=0.605; *P*=.59; mean pretest score 8.80, SD 4.75; mean posttest score 9.33, SD 3.83), magnification (*Z*=–0.247; *P*=.84; mean pretest score 5.27, SD 2.02; mean posttest score 5.13, SD 3.09), or total (*Z*=–1.224; *P*=.24; mean pretest score 25.60, SD 11.48; mean posttest score 23.60, SD 11.02). These results suggest that 8 sessions of KVET are an effective treatment for improving the feeling of helplessness associated with chronic pain and allows a person to experience that they can influence pain and movement.

**Figure 2:**
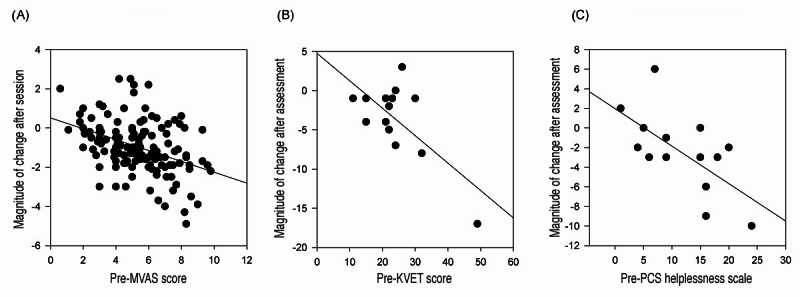
The magnitude of improvement on measurements that showed a significant improvement. (A) Magnitude of change for MVAS; (B) magnitude of change for Oswestry; and (C) magnitude of change for PCS helplessness. PCS: Pain Catastrophizing Scale; KVET: Karuna Virtual Embodiment Training; MVAS: mechanical visual analog scale.

We analyzed the magnitude of change in scores before and after training to assess the degree to which patients with chronic pain improved on pain intensity before and after each session, low back pain disability rating before and after 8 KVET sessions, and the sense of helplessness in dealing with pain before and after 8 KVET sessions. The magnitude was defined as the amount of reduction between pre- and posttraining scores. Figure 2 displays scatter plots of the magnitude of change of the pre- (x-axis) and posttraining (y-axis) scores. A Pearson product correlation analysis revealed a significant correlation for VAS (*r*=–0.41; *P*<.001), PCS helplessness (*r*=–0.64; *P*=0.01), and ODI (*r*=–0.69; *P*=.003).

## Discussion

### Principal Findings

This study aimed to assess the feasibility and safety of a novel digital therapy that delivers functional rehabilitation through virtual embodiment in VR. Patient-reported exploratory measures were administered to assess the effectiveness of virtual embodiment training as a viable nonpharmacological, noninvasive treatment of pain. Pain intensity, as measured by the VAS, showed statistically significant reductions after each individual KVET session but no improvement session over session. Disability, as measured by the ODI, and pain catastrophizing all improved as a function of a 4-week virtual embodiment training.

In this experiment, pain intensity was reduced after the intervention using embodiment in VR. The effect size was similar to that reported in magnetic neural stimulation for chronic pain in spinal cord injury [68] and acceptance and commitment therapy to treat chronic pain [69], suggesting that this protocol is a promising intervention for patients with chronic pain. The use of VR in interventions has been previously proposed to be an interesting nonpharmacological alternative for treating chronic pain [70]. VR technology is rapidly advancing due to the introduction of headsets such as Oculus Rift (Meta) and HTC Vive. The recent advances in handheld controllers, cameras, and body trackers have enabled first-person body experience in VR. Embodiment (sometimes referred to as immersiveness) is the process by which a person identifies with and develops a sense of ownership over a body part that is not their own. In this experiment, virtual embodiment, the first-person perception of sensory feedback of the actions of an avatar, was used. Using VR, it is possible to display movements that are not possible for the patient in physical reality and modify the movement to appear more understated or exaggerated. When a person experiences embodiment, sensorimotor representation in the central nervous system of the painful limb is suggested to go through a reorganization. This modification in the representation and thus the perception of a painful limb allows people to perceive the limb as less painful [48,53-55]. Although the sense of embodiment was not measured in this feasibility study, the reduction in the pain intensity observed in this study before and after each intervention session could be due to the embodiment protocol used, as embodiment has the potential to shift the perception of pain to less intense and less threatening.

The reduction of pain in this experiment could also be partially due to the specific exercises used. Exercises used in this study’s intervention through KVET were designed based on the principles of GMI. GMI is a biopsychosocial approach to physical therapy aiming for a gradual reintroduction of motor actions and was developed as a systematic approach to ready subjects with severe chronic pain to engage their sensorimotor pathways related to their painful bodily regions. In a meta-analysis of 6 randomized control trials, GMI was found to produce substantial pain reduction in chronic pain conditions of the upper limbs, more efficiently than usual physiotherapy [71]. GMI consists of 3 stages: (1) *laterality testing*, in which the participant identifies whether a limb is the right or left limb; (2) *motor imagery*, in which the participant imagines the movement of his or her affected limb while engaging motor pathways to “feel” as though they are actually moving; and (3) *mirror therapy*, or the mirror visual feedback (MVF) [72]. In this experiment, MVF was incorporated into the VR experience for treating chronic pain. Initially developed to treat phantom limb pain, MVF is a dynamic therapy that uses a mirror image of an unaffected contralateral limb to visually represent the function and motion of an affected or impaired limb. MVF has been shown effective in treating complex regional pain syndrome in randomized control trials, which found clinically significant reduction in pain above conventional physical therapy techniques [73]. The application of MVF through the virtual embodiment could have reinforced its effectiveness in this study. However, although pain intensity was reduced after each session, there was no change in pain intensity over the course of 4 weeks. Previously, GMI and similar interventions that aim for cortical remapping for neuroadaptive changes in chronic lower back pain have found short-term benefits on pain intensity and disability while long-term effects are yet to be confirmed [74]. The VR intervention in this experiment was designed based on GMI, suggesting that GMI through VR shows similar short-term benefits. Alternatively, the lack of reduction in the pain intensity over the course of 4 weeks could also be due to the number of sessions. Perhaps more durable improvements in pain intensity requires more sessions for attaining adaptive neuroplasticity in chronic pain. Future studies with more frequent sessions or longer interventions using VR could clarify the lack of long-term effects in this study.

In this study, the disability component of the ODI and the helplessness subscale of the PCS were reduced over the course of 4-week intervention, suggesting that the virtual embodiment protocol used was effective in improving psychological measures. Moreover, the observed effect size of reduction in disability was similar in magnitude to that reported for other nonsurgical treatments for chronic pain such as acupuncture, behavioral therapy, and exercise [75], suggesting that 8 sessions of KVET is an effective treatment for chronic low back pain disability. Similarly, the helplessness was also reduced over the course of 4-week immersiveness intervention, suggesting that 8 sessions of KVET are an effective treatment for improving the feeling of helplessness associated with chronic pain. Previously, digital rehabilitation therapy has been shown to reduce disability while increasing the range of motion in patients with chronic cervical neck pain [58]. Digital rehabilitation therapy was suggested to alter the activation of pain pathways in the central nervous system [76] with 2 approaches of distraction and immersiveness [48,51]. In this experiment, through the embodiment, the gradual exposure of patients could have helped the patients overcome learned behaviors and fear avoidance due to chronic pain. The immersiveness in a VR setting could be especially pertinent for individuals with chronic pain who experience reduced motivation for volitional movement. Previously, similar rehabilitation sessions of Fear Avoidance Beliefs Training have shown improvements in pain catastrophizing, pain-related mobility impairment, and pain intensity and disability [49]. Moreover, in this experiment, there was a negative correlation between the change over the course of 4 weeks in pain intensity and helplessness as well as pain intensity and disability, suggesting that the patients with the most severe pain may benefit the most from KVET therapy on psychological measures related to chronic pain. The immersive VR experience could have allowed the person to gain a sense of control and belief that they can influence the pain and improve movement. With improved movement, in turn, the brain could have dampened the threat response to movement. Thus, over time, adaptive neural pathways are established, allowing the patients to be desensitized to pain associated with movement.

Although this study provides evidence for safety and the benefits of a 4-week virtual embodiment training for chronic pain, there were also limits. Notably, the lack of a control group is an important limitation of this study. Further studies are to be carried with a control group who is following the standard of care for chronic pain. Additionally, this study did not have a follow-up measure, hence the long-term efficiency and effects of the treatment are not known.

Finally, in this experiment, all 24 patients completed all planned sessions during the 4-week virtual embodiment training without adverse events, suggesting that when designed correctly, VR therapies that combine pain relief and movement may provide nonpharmacological, noninvasive, and nonaddiction modalities for treating chronic pain. This study’s results support the justification for a larger randomized controlled trial to assess the extent to which virtual embodiment training can exert an effect on symptoms associated with chronic pain. VR may be ubiquitous in homes within the near future, this could introduce the possibility of engaging in neurorehabilitation from the convenience of the home, with data from sessions accessible by prescribing clinicians. Further research is needed to explore the potential use and effects of VR for managing chronic pain.

### Conclusion

This experiment showed that the gradual exposure of patients to functional rehabilitation through a rehabilitation medical device provided cognitive retraining, improving pain intensity after each session as well as pain-related disability and helplessness over the course of 4 weeks. When designed correctly, VR therapies that combine pain relief and movement may provide nonpharmacological, noninvasive, and nonaddiction modalities for treating chronic pain.

## Appendix

Multimedia Appendix 1 CONSORT eHEALTH Checklist (V 1.6.1).

## Abbreviations

ACC: anterior cingulate cortex
FABQ: Fear-Avoidance Beliefs Questionnaire
GMI: graded motor imagery
KVET: Karuna Virtual Embodiment Training
MVF: mirror visual feedback
ODI: Oswestry Disability Index
PAG: periaqueductal gray
PCS: Pain Catastrophizing Scale
SSQ: Simulator Sickness Questionnaire
VAS: visual analog scale
VR: virtual reality
